# Identification of Quantitative Trait Loci Controlling High-Temperature Tolerance in Cucumber (*Cucumis sativus* L.) Seedlings

**DOI:** 10.3390/plants9091155

**Published:** 2020-09-07

**Authors:** Shaoyun Dong, Song Zhang, Shuang Wei, Yanyan Liu, Caixia Li, Kailiang Bo, Han Miao, Xingfang Gu, Shengping Zhang

**Affiliations:** Institute of Vegetables and Flowers, Chinese Academy of Agricultural Sciences, Beijing 100086, China; dongshaoyun@caas.cn (S.D.); song.zhang@bioseed.cn (S.Z.); 82101181120@caas.cn (S.W.); 82101182238@caas.cn (Y.L.); 82101182236@caas.cn (C.L.); bokailiang@caas.cn (K.B.); miaohan@caas.cn (H.M.)

**Keywords:** cucumber seedlings, thermotolerance, QTL mapping

## Abstract

High temperature is one of the major abiotic stresses that affect cucumber growth and development. Heat stress often leads to metabolic malfunction, dehydration, wilting and death, which has a great impact on the yield and fruit quality. In this study, genetic analysis and quantitative trait loci (QTL) mapping for thermotolerance in cucumber seedlings was investigated using a recombinant inbred line (RILs; HR) population and a doubled haploid (DH; HP) population derived from two parental lines ‘65G’ (heat-sensitive) and ‘02245′ (heat-tolerant). Inheritance analysis suggested that both short-term extreme and long-term mild thermotolerance in cucumber seedlings were determined by multiple genes. Six QTLs for heat tolerance including *qHT3.1*, *qHT3.2*, *qHT3.3*, *qHT4.1*, *qHT4.2*, and *qHT6.1* were detected. Among them, the major QTL, *qHT3.2*, was repeatedly detected for three times in HR and HP at different environments, explained 28.3% of the phenotypic variability. The 481.2 kb region harbored 79 genes, nine of which might involve in heat stress response. This study provides a basis for further identifying thermotolerant genes and helps understanding the molecular mechanism underlying thermotolerance in cucumber seedlings.

## 1. Introduction

Cucumber (*Cucumis sativus* L.) is an economically important crop grown all over the world. Global production of cucumber reached 75.2 million metric tons, 75.2% of which was produced in China [1]. Cucumber originates from tropical regions, and the suitable temperature for its growth is 18–30 °C. However, with the acceleration of the greenhouse effect, the temperature is gradually increasing worldwide, which greatly restricts the cucumber annual cultivation [2,3]. In China, cucumber often experiences heat stress, especially in the late spring and early autumn of facility cultivation, where the temperature often exceeds 35 °C and could reach 50 °C [4]. Long-term high temperature above 40 °C often results in metabolic malfunction, water loss and wilting of cucumber, and short-term extreme high temperature above 50 °C leads to macro-molecule degradation, cell structure damage, dehydration and death, which has a great impact on the yield and quality of cucumber [2].

Thermotolerance in cucumber is determined by multiple alleles. Using the heat-tolerant line R1 and heat-sensitive line R29, it was found that heat tolerance fits an additive-dominant model, mainly with additive effect [5]. Xu [6] used a major gene-polygene mixed genetic model to analyze the thermotolerance of cucumber seedling, and found that it was controlled by two major genes with addictive-dominant-epistasis effect and polygenes with addictive-dominant effects. Zhang [7] found that the thermotolerance of cucumber was determined by major genes with addictive effect.

Few studies on quantitative trait loci (QTL)/gene mapping of thermotolerance have been reported in cucumber. Yang [8] identified one SSR marker related to cucumber thermotolerance using BSA method. Yang [9] identified four SSR and seven RAPD markers related to thermotolerance, using relative conductivity as an indicator. Chen et al. [10] used the relative growth as the index and identified 16 markers that were linked to heat tolerance loci; Zhuang [11] used F_2_ as the mapping population, and detected one QTL on chromosome 5, which explained 11.0% of the phenotypic variation.

Plants activate a series of genes to cope with heat stress, including genes encoding heat shock proteins (Hsps), heat stress transcription factors (Hsfs), and those involved in antioxidation, maintenance of membrane stability, and hormone signaling pathway [12]. Studies have showed that cucumber heat shock protein *CsHSP70* was highly expressed in both heat-tolerant and heat-sensitive lines under heat stress [13]. The expression patterns of Hsfs in cucumber were studied and most of them were up-regulated under heat stress, indicating those involved in heat stress tolerance [14]. There are also studies showed that auxin synthesis genes *CsYUC8* and *CsYUC9* were significantly up-regulated in heat-tolerance materials [15], and overexpressing *CsCaM3*, a Calmodulin gene, could prevent peroxidation and photosynthesis system damage, and thus improve heat tolerance [16].

The objective of this study was to identify QTLs responsible for thermotolerance in cucumber seedlings using recombinant inbred line (RIL) and double haploid (DH) populations constructed from heat tolerant inbred line 02245 and heat sensitive inbred line 65G. The results from this study will promote the breeding of thermotolerant cucumber varieties and help the understanding of the molecular mechanism underlying thermotolerance in cucumber seedlings.

## 2. Results

### 2.1. Characterization of Thermotolerance in Cucumber Seedlings

**Figure 1:**
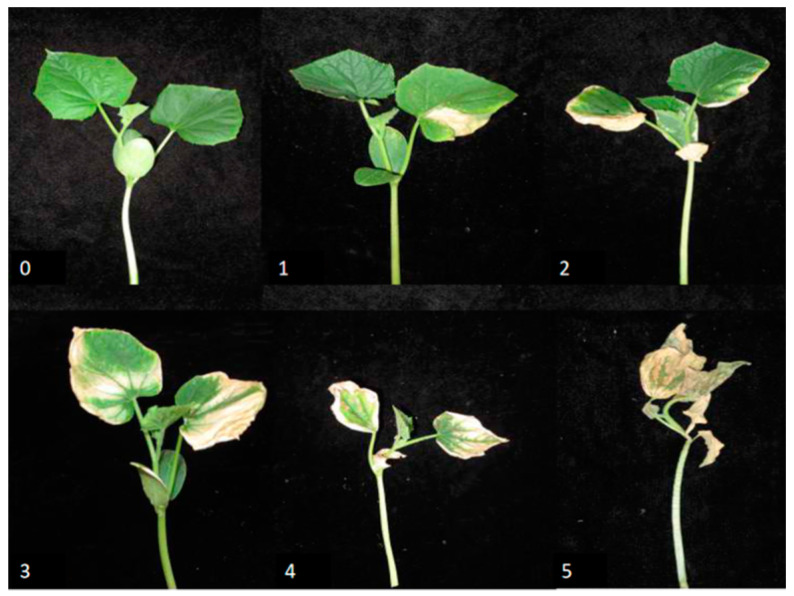
Phenotypic characterization of thermotolerance in cucumber seedlings. Heat injury index (HII) was used to indicate thermotolerance based on the dehydration of the two mature leaves and young leaf. 0: no symptoms on both the mature and young leaves; 1: the young leaf has no symptoms, while less than 1/3 of the mature leaves were dehydrated; 2: the young leaf has few dehydrated spots, while 1/3–2/3 of the mature leaves were dehydrated; 3: less than 1/2 of the young leaf was dehydrated, while more than 2/3 of the mature leaves were dehydrated; 4: more than 1/2 of the young leaf was dehydrated, and more than 2/3 of the mature leaves were dehydrated; 5: both the young and mature leaves were dehydrated.

To analyze the inheritance pattern of thermotolerance in cucumber seedlings, HR, which is a 140 RIL population generated from inbred lines ‘65G’ (heat sensitive) × ‘02245’ (heat tolerant), together with HP, which is a doubled diploid population generated from F_1_ (‘65G’ × ‘02245’) embryo culture, were used for heat tolerance characterization. Two-mature-leaf stage seedlings of the two parental lines, F1, HR, HP populations were exposed to short-term extreme heat stress (50 ± 2 °C for 3.5 h) at Shunyi, Beijing (SY), and long-term mild heat stress (1.5–6 h of daily temperature above 40 °C for 9 d) at Nankou, Beijing (NK). After the stress treatment, the heat injury was classified into six grades, based on the dehydration degree of the two mature leaves and young leaf (Figure 1).The heat injury index (HII) was used to indicate the thermotolerance performance of each plant.

**Figure 2:**
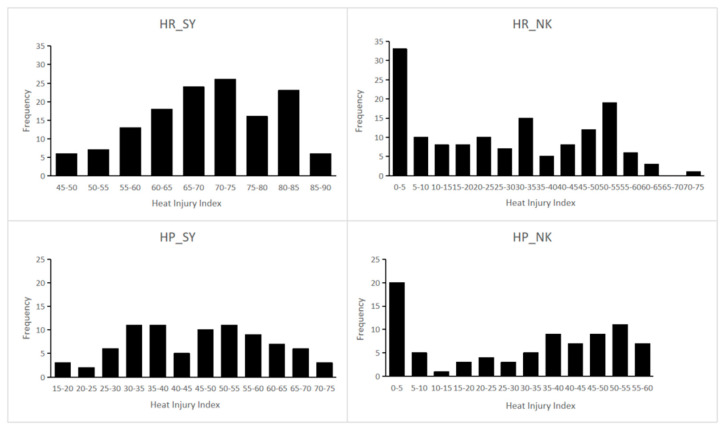
Frequency distribution of HII in RILs and DH populations. HR_SY, HR_NK, HP_SY, HP_NK indicate HII data collected from two populations (HR and HP) exposed to short-term extreme heat stress (SY = Sunyi, Beijing) and long-term mild heat stress (NK = Nankou, Beijing), respectively. X axis indicates the heat injury index (HII), Y axis indicates the number of individuals in each HII category.

For the experiment conducted in the greenhouse at SY, after the seedlings were exposed to heat shock (50 ± 2 °C) for 3.5 h, the first two mature leaves and the young leaf of ‘65G’ all showed severe dehydration, while ‘02245’ showed slight symptoms. The HII of ‘65G’ and ‘02245’ were significantly different, which was 76.9 and 24.5, respectively. The HII of the F_1_ hybrids was 44.8, with the performance more inclined to ‘02245’ (Table 1). Frequency distribution of the HII among the HR and HP population showed continuous variation from tolerance to sensitive phenotypes (Figure 2).

For the experiment conducted in greenhouse at NK, the seedlings were exposed to long-term mild heat stress (1.5–6 h of daily temperature above 40 °C for 9 d). After ten days of stress treatment, ‘65G’ and ‘02245’ showed significant differences in thermotolerance, with HII of 42.5 and 23.0, respectively. The HII of F_1_ hybrids was 30.7, which is more close to 02245 as well (Table 1). Compared with the HII of individuals exposed to heat shock, the HII of HR and HP populations under long-term mild heat stress were generally lower, many individuals had slight dehydration symptoms with HII of 0–5, and the others were continuously distributed within 5 to 75 (Figure 2).

In summary, the above two experiments suggested that short-term extreme and long-term mild heat tolerance of line ‘02245’ is a quantitative trait that is controlled by multiple genes.

### 2.2. QTL Analysis of Thermotolerance in Cucumber Seedlings

To detect QTLs for thermotolerance in cucumber seedlings, the phenotypic data for thermotolerance (HII) from HR and HP in two environments (Supplementary Materials Tables S1 and S2) and their genetic maps that we previously constructed [17] were used for QTL mapping, respectively. Details of each QTL detected, including chromosome number, physical position, length of interval, peak logarithm of odds (LOD) support value, and percentages of total phenotypic variances explained (R^2^) are shown in Table 2.

**Figure 3:**
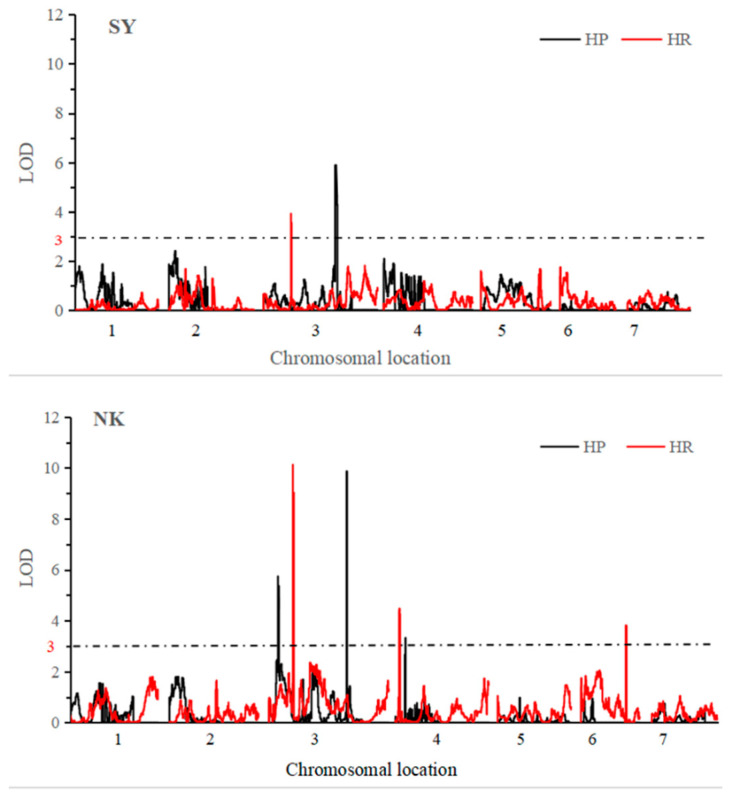
QTL analysis of thermotolerance in cucumber seedlings. The x-axis indicates the genetic position of each chromosome, the y-axis indicates the LOD value. SY: experiment conducted at Sunyi, where seedlings were exposed to short-term extreme heat stress, NK: experiment conducted at Nankou, where seedlings were exposed to long-term mild heat stress.

For the short-term extreme heat stress treatment at SY, one QTL was detected using the HR and HP population, respectively (Figure 3). One QTL on Chr.3 named *qHT3.1* (Chr.3: 31,236,735–31,344,231) was detected using HR, with LOD score of 3.9 and explained 12.2% of the phenotypic variation. One QTL on Chr.3 named *qHT3.2* (Chr.3: 31,699,402–33,960,084) was detected in HP, with a LOD score of 5.9 and explained 27.6% of the phenotypic variation. These two loci were physically close with each other, they thus might be the same locus.

For the long-term mild heat stress treatment at NK, three QTLs including *qHT3.2* (Chr.3: 32,180,242–32,661,417) on Chr.3, *qHT4.1* (Chr.4: 21,608,172–21,864,140) on Chr.4 and *qHT6.1* (Chr.6: 4,439,107–4,498,675) on Chr.6 were detected in HR (Figure 3). Among them, *qHT3.2* had the highest LOD score of 10.1, and accounts for 28.3% of the phenotypic variation. Moreover, three QTLs including *qHT3.3* (Chr.3: 4,939,077–5,566,247) and *qHT3.2* (Chr.3: 31,776,615–32,667,875) on Chr.3, and *qHT4.2* (Chr.4: 18,727,523–18,935,103) on Chr.4 were detected in HP. Among them, *qHT3.2* had a LOD score of 9.9, and accounts for 26.5% of the phenotypic variation.

In summary, six QTLs in total were identified using two populations exposed to short-term extreme heat stress and long-term mild heat stress. *qHT3.1* and *qHT3.2*, which were physically close with each other (350 Kb away), might be the same locus. Besides, major effect QTL *qHT3.2* was repeatedly detected three times using two populations in two stress treatments, and it was thus considered as a major QTL that is responsible for both short-term extreme and long-term mild thermotolerance in cucumber seedlings. In addition, two QTLs on Chr.4 named *qHT4.1* (Chr.4: 21,608,172–21,864,140) and *qHT4.2* (Chr.4: 18,727,523–18,935,103) were detected in HR and HP populations under long-term mild heat stress. Interestingly, we previously performed genome-wide association study analysis to detect signals related to thermotolerance using 86 cucumber core germplasms, and identified two signals on Chr.4 named *gHII4.1* (Chr.4: 17,294,615–18,294,615) and *gHII4.2* (Chr.4: 17,744,278–18,744.278) (Wei et al. 2019). It is worth noting that *qHT4.2* identified in this study is very close to *gHII4.1* and *gHII4.2*, and they thus might be the same locus.

### 2.3. Candidate Gene Analysis of Heat Stress Tolerance within Major QTL qHT3.2

Since the 481.2-Kb region within *qHT3.2* was repeatedly detected three times in HR and HP at two environments, we proposed that it plays an important role in both short-term extreme and long-term mild thermotolerance of cucumber seedlings. Based on Chinese Long V2.0 genome (http://cucurbitgenomics.org/organism/2), this region contains 79 annotated genes (listed in Supplementary Materials Table S3), and gene annotation suggested that nine of them might be the candidate genes (Table 3). They are genes encoding chaperone (*Csa3G822410*) that directly involves in heat tolerance, and stress responsive proteins like nucleotide binding site (NBS)-containing resistance-like proteins (*Csa3G824920*, *Csa3G824940*) and Calmodulin-like protein 1 (*Csa3G823060*, *Csa3G825010*), and five stress responsive transcription factors.

**Figure 4:**
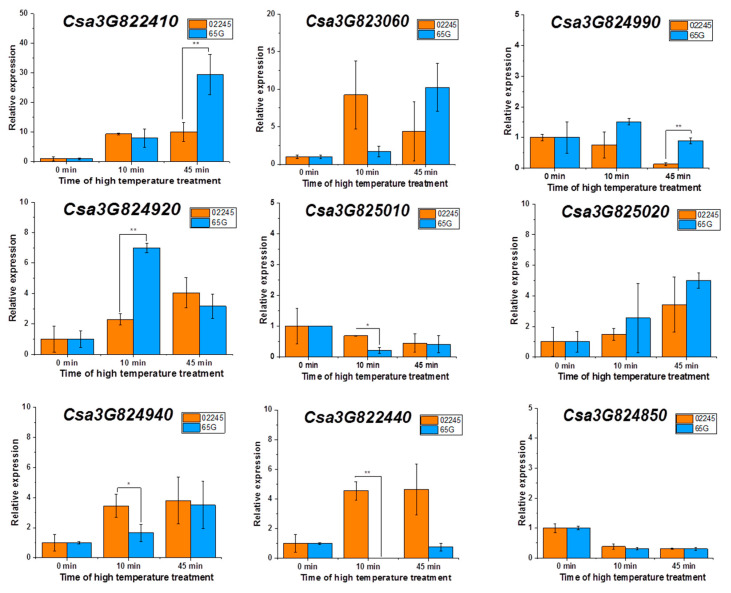
Quantification of transcripts from the candidate genes involve in thermotolerance. The transcript levels of genes at 0 min were set as 1. The x-axis indicates the time after 50 °C heat stress exposure, the y-axis indicates the relative amount of gene transcripts at different time-points compared to 0 min. RNA samples were extracted from two-leaf-stage seedlings that were exposed to 50 °C heat stress for 0, 10, and 45 min. Error bars represent the standard deviation. The asterisks indicate that there were significant differences in transcript level between heat-tolerant cucumber line ‘02245’ and heat-sensitive cucumber line ‘65G’ (‘*’, 0.01 < *p* < 0.05; ‘**’, 0.001 < *p* < 0.01).

To further investigate the differential expression pattern of candidate genes in heat-tolerant line ‘02245’ and heat-sensitive line ‘65G’ under heat stress, the two-leaf stage seedlings were exposed to 50 °C heat stress for 0, 10, 45 min, and the dynamic expression changes of the nine candidate genes were evaluated. As Figure 4 shows, 10 min after heat stress treatment, a significantly different transcript level of *Csa3G824920*, *Csa3G824940*, *Csa3G825010*, and *Csa3G822440* was detected. Among them, the transcript levels of *Csa3G824940*, *Csa3G825010*, and *Csa3G822440* were significantly higher in heat-tolerant line ‘02245’, indicating they might play positive role in early heat stress response. At 45 min after heat stress, the expression level of *Csa3G822410* and *Csa3G824990* were significantly higher in heat-sensitive line ‘65G’. These genes could be further investigated to isolate the target gene that is responsible for cucumber thermotolerance.

## 3. Discussion

In our study, the thermotolerance in cucumber seedlings was characterized using two populations (HR and HP) in two environments (short-term extreme heat stress and long-term mild heat stress). Using the dehydration degree of the two mature leaves and young leaf as the heat injury index, inheritance analysis suggested that thermotolerance in cucumber seedlings is a quantitative trait that is controlled by multiple genes. Several studies on inheritance analysis of heat stress have been previously reported, however the results were different due to the variation of plant materials used, differences in treatment conditions, and various evaluation standards. It was reported that using heat-tolerant line R1 and heat-sensitive lines R29, it was found that the heat tolerance fits an additive-dominant model, mainly with additive effect [5]. There are also studies that showed that heat tolerance was controlled by two major genes with addictive-dominant-epistasis effect and polygene with addictive-dominant effect [6], and multiple major genes with addictive effect [7].

Six QTLs were identified in different environments using two populations. In addition, *qHT3.1* (Chr.3: 31,236,735–31,344,231) and *qHT3.2* (Chr.3: 31,699,402–33,960,084), *qHT4.1* (Chr.4: 21,608,172–21,864,140) and *qHT4.2* (Chr.4: 18,727,523–18,935,103) were closely located, and might be the same locus. *qHT3.2* was identified three times in two populations under both extreme and mild heat stress conditions, and this suggests that *qHT3.2* plays an important role in thermotolerance in cucumber seedlings. Few studies on QTL/gene mapping of heat tolerance in cucumber have been reported so far. Zhuang [11] characterized the heat resistance of the six generations and used F_2_ as the mapping population, and detected one QTL on Chr. 5, which explained 11.0% of the phenotypic variation. Previous study on the heat tolerance of 86 cucumber core germplasms identified two loci *gHII4.1* (Chr.4: 17,294,615–18,294,615) and *gHII4.2* (Chr.4: 17,744,278–18,744.278) associated with heat tolerance by genome-wide association study analysis (Wei et al. 2019). It is worth noting that *qHT4.2* identified in our study was very close to *gHII4.1* and *gHII4.2*, indicating this region might contain thermotolerant genes that need further investigation.

The physical length of the *qHT3.2* region was 481.2 kb (31,699,402–33,960,084 bp), harboring 79 genes. Within this region, *Csa3G822410* that encodes a heat shock protein was identified. Heat shock proteins (Hsps) function as molecular chaperones, and are major functional proteins that are induced by heat stress [18,19]. Plants accumulate Hsps/chaperones to improve thermotolerance through maintaining proteins in their functional conformations and preventing the aggregation of non-native proteins, thus re-establishing cellular homeostasis [20,21,22,23,24,25]. In cucumber, Hsp gene *CsHsp45.9* was found harbor broad-spectrum responses to both biotic and abiotic stresses [26], and *CsHsp70* was significantly induced by heat in two thermotolerant cucumber lines [13]. Besides, *Csa3G824920* and *Csa3G824940* that encode NBS-containing resistance-like proteins, and *Csa3G825010* that encodes Calmodulin-like protein 1, were also found within this region. A previous study suggested that overexpressing *CsCaM3*, a Calmodulin gene, could prevent peroxidation and photosynthesis system damage, and thus improve heat tolerance [16]. Two transcription factors including Csa3G824990 and Csa3G822440 that encodes an NAC domain protein and an AP2-like ethylene-responsive transcription factor were also found in the major QTL. Transcription factors (TFs) play an essential role in plants abiotic stress response by activating the expression of target genes [27]. The genome-wide identification and phylogenetic analysis of NAC and ERF gene families have been reported in cucumber [28,29,30,31], however, the role of these TFs in thermotolerance was not functionally characterized. The candidate genes identified in this study could be further studied to understand their molecular mechanism underlying thermotolerance in cucumber seedlings.

## 4. Materials and Methods

### 4.1. Plant Materials

The lines ‘65G’ and ‘02245’ are two inbred lines of cucumber (Cucumis sativus L.), which have high sensitivity and tolerance to heat stress, respectively. Two populations were used in QTL mapping for heat stress tolerance, including 140 recombinant inbred lines (RILs) from 65G × 02245 (HR hereafter), and 85 DH populations (HP hereafter) generated from Megaspore culture. All materials were provided by the Cucumber Genetic Breeding Group, Institute of Vegetables and Flowers, Chinese Academy of Agricultural Sciences (CAAS).

### 4.2. Phenotypic and Genetic Analysis of Thermotolerance

Heat stress treatments were carried out in the plastic greenhouse of the IVF, CAAS at Shunyi, Beijing (SY) and Nankou, Beijing (NK), respectively. At SY, two-mature-leaf stage seedlings of two parental lines, F1, HR, and HP populations were transported to a plastic greenhouse (50 ± 2 °C) for 3.5-h heat shock. At NK, two-mature-leaf stage seedlings were exposed to moderate heat stress (1.5–6 h of daily temperature above 40 °C) for 9 d. In both environments, the high temperature was naturally happed during summer in cucumber production. The detailed real-time temperature and humidity during the heat stress treatment was recorded and listed in Supplementary Materials Tables S4 and S5. After the stress treatments, phenotypic data of the heat injury in HR and HP were collected, and the heat injury of each plant was divided into six grades, according to the dehydration degree of two mature leaves and young leaf (Figure 1). The six grades were listed as follows: 0: no symptoms on neither the mature nor young leaves; 1: the young leaf was normal, while less than 1/3 of the mature leaves was dried; 2: the young leaf has little dryness spots, while 1/3–2/3 of the mature leaves were dried; 3: less than 1/2 of the young leaf was dried, while more than 2/3 of the mature leaves were dried; 4: more than 1/2 of the young leaf was dried, and more than 2/3 of the mature leaves were dried; 5: both the young and mature leaves were dried. A heat injury index (HII) was used to indicate the thermotolerance of each plant. The formula below was used to calculate the HII as previously used [32]: HII = ((0 × S0 + 1 × S1 + 2 × S2 + 3 × S3 + 4 × S4 + 5 × S5)/5 × N) × 100. S0–S5 indicates the number of plants divided into each grade. N indicates the total number of plants. Three replicates were set for each treatment, and seven plants were investigated for each replicate.

### 4.3. QTL Analysis of Heat Tolerance in HR and HP

Genetic maps of HR and HP that were previously generated in our lab [17] were employed for QTL analysis in this study. QTL analysis was performed as previously described in our lab [33]. Briefly, an interval mapping (IM) analysis was performed to identify QTLs using MapQTL 4.0 [34]. The LOD threshold values were determined by computing 1000 permutations at the *p* = 0.05 level. The possibility of QTL existence was scanned on each chromosome at intervals of 1 cM. QTLs that were detected were verified by the multiple-QTL model (MQM). For each QTL, a 2-LOD interval was calculated. The QTL naming format refers to a previous study [35].

### 4.4. Real-Time Quantitative Reverse Transcription PCR (qRT-PCR) Analysis

The seeds of ‘65G’ and ‘02245’ were sown in pots (12 × 12 × 10.8 cm) containing nutrient soil in growth chamber with day temperature of 28 °C and night temperature of 20 °C. The two-leaf stage seedlings were exposed to 50 °C heat stress when they were two leaves old. The second true leaf of each plant was sampled at 0, 10, 45 min after heat stress, respectively, for RNA extraction. The qPCR primers used were listed in Supplementary Materials Table S6. The qRT-PCR was performed using SYBR Premix Ex TaqTM (Tli RNaseH Plus) (Takara Bio Inc., Beijing, China) in Roche Diagnostics with Light Cycler 480 System. *Actin1* (*Csa3G806800*) was the reference gene used for normalizing gene expression values [36]. Three biological replicates were set for each treatment and three technical replicates were performed. The relative expression level of candidate gene was calculated using the 2^−ΔΔCt^ method [37].

### 4.5. Statistical Analysis

The significant differences between ‘02245’ and ‘65G’ were analyzed using one-way ANOVA in the R environment (R Development Core Team 2006).

## 5. Conclusions

We reported that the short-term extreme and long-term mild thermotolerance in cucumber seedlings is a quantitative trait that is controlled by multiple genes. Two loci, namely *qHT3.1* and *qHT3.2*, and five loci, namely *qHT3.2*, *qHT3.3*, *qHT4.1*, *qHT4.2*, and *qHT6.1* that regulate short-term extreme or long-term mild thermotolerance in cucumber seedlings, were detected. Furthermore, within the major QTL *qHT3.2*, which was repeatedly detected in two stress environments using two populations, candidate genes that are involved in the heat stress response were predicted. This study lays a foundation for identifying genes responsible for thermotolerance and helps understanding of the molecular mechanism underlying heat tolerance in cucumber seedlings.

## Figures and Tables

**Table 1:** Frequency distribution of HII in recombinant inbred lines (RILs) and double haploid (DH) populations.

		Parental Line		F_1_	RILs or DH Population				
Population	Location	‘65G’	‘02245’		Means	SD	CV	Kurtosis	Skewness
HR (RILs)	SY	76.9 ± 1.7 **	24.5 ± 4.5	44.8	68.3 ± 1.5	17.6	0.3	59.8	−6.4
	NK	42.5 ± 2.5 *	23.0 ± 1.5	30.7	26.8 ± 1.8	21.4	0.8	−1.4	0.1
HP (DH)	SY	76.9 ± 1.7 **	24.5 ± 4.5	44.8	46.0 ± 1.3	13.8	0.3	−0.6	−0.1
	NK	42.5 ± 2.5 *	23.0 ± 1.5	30.7	27.5 ± 2.0	21.4	0.8	−1.6	−0.1

Note: SY: experiment conducted at Sunyi, where seedlings were exposed to short-term extreme heat stress (50 ± 2 °C for 3.5 h), NK: experiment conducted at Nankou, where seedlings were exposed to long-term mild heat stress (1.5–6 h of daily temperature above 40 °C for 9 d), SD indicates standard deviation, CV indicates coefficient of variation, * indicates that the value is significant at *p* ≤ 0.05; ** indicates that the value is significant at *p* ≤ 0.01.

**Table 2:** QTLs for thermotolerance that were identified in RIL and DH populations in two heat stress treatments.

Treatments	Populations	QTL	Chr.	Physical Pos.	Length (Kb)	Peak LOD	Exp%
SY	HR	*qHT3.1*	3	31236735–31344231	107.5	3.9	12.2
	HP	*qHT3.2*	3	31699402–33960084	2260.7	5.9	27.6
NK	HR	*qHT3.2*	3	32180242–32661417	481.2	10.1	28.3
	HR	*qHT4.1*	4	21608172–21864140	256	4.5	13.7
	HR	*qHT6.1*	6	4439107–4498675	59.6	3.8	11.8
	HP	*qHT3.3*	3	4939077–5566247	627.2	5.7	13.1
	HP	*qHT3.2*	3	31776615–32667875	891.3	9.9	26.5
	HP	*qHT4.2*	4	18727523–18935103	207.6	3.3	16.6

**Table 3:** Annotation of candidate genes involve in thermotolerance within major QTL *qHT3.2*.

Gene ID	Annotation
*Csa3G822410*	HSP20-like chaperone
*Csa3G824920*	NBS-containing resistance-like protein
*Csa3G824940*	TIR-NBS-LRR resistance protein
*Csa3G823060*	Calmodulin-like protein 1
*Csa3G825010*	Calmodulin-like protein
*Csa3G824990*	NAC domain protein
*Csa3G822440*	AP2-like ethylene-responsive transcription factor
*Csa3G825020*	Dof zinc finger protein
*Csa3G824850*	MYB transcription factor

## Supplementary Materials

The following are available online at https://www.mdpi.com/2223-7747/9/9/1155/s1, Table S1: Phenotypic data of thermotolerance in HR population, Table S2: Phenotypic data of thermotolerance in HP population, Table S3: Candidate genes within the major QTL qHT3.2, Table S4: Heat stress treatment conducted at Shunyi, Beijing (SY), Table S5: Heat stress treatment conducted at Nankou, Beijing (NK), Table S6: Oligos used for qRT-PCR.

## Abbreviations

QTL	quantitative trait loci
RIL	recombinant inbred line
DH	double haploid
HII	heat injury index
SY	Shunyi
NK	Nankou
